# Both Alcoholic and Non-Alcoholic Liver Cirrhosis Are Associated with an Increased Risk of HF—A Cohort Study Including 75,558 Patients

**DOI:** 10.3390/jcdd12080295

**Published:** 2025-07-31

**Authors:** Karel Kostev, Jamschid Sedighi, Samuel Sossalla, Marcel Konrad, Mark Luedde

**Affiliations:** 1 IQVIA, Epidemiology, 60549 Frankfurt am Main, Germany; 2 University Hospital, Philipps University of Marburg, 35043 Marburg, Germany; 3 Medical Clinic I, Cardiology and Angiology, University Hospital of Giessen and Marburg, Campus Giessen, 35392 Giessen, Germany; 4 Department of Cardiology, Kerckhoff-Clinic, 61231 Bad Nauheim, Germany; 5 German Center for Cardiovascular Research (DZHK), Partner Site Rhine-Main, 61231 Bad Nauheim, Germany; 6 Health & Social, FOM University of Applied Sciences for Economics and Management, 60486 Frankfurt am Main, Germany; 7 Christian-Albrechts-University of Kiel, 24118 Kiel, Germany

**Keywords:** heart failure, liver cirrhosis, association, risk factor, Germany

## Abstract

The objective of the present study was to evaluate the association between liver cirrhosis (LC) and subsequent Heart failure (HF). This retrospective cohort study utilized data from the Disease Analyzer database (IQVIA) and included adults with a first-time diagnosis of LC in 1293 general practices in Germany between January 2005 and December 2023. A comparison cohort without liver diseases was matched to the cirrhosis group using 5:1 propensity score matching. Univariable Cox proportional hazards models were used to assess the association between alcoholic vs. non-alcoholic LC and HF. The final study cohort included 5530 patients with alcoholic LC and 27,650 matched patients without liver disease, as well as 7063 patients with non-alcoholic LC and 35,315 matched patients without liver disease. After up to 10 years of follow-up, HF was diagnosed in 20.9% of patients with alcoholic LC compared to 10.3% of matched cohort, and in 23.0% of patients with non-alcoholic LC, compared to 14.2% in matched cohort. Alcoholic LC (Hazard Ratio (HR): 2.07 (95% CI: 1.85–2.31) and non-alcoholic LC (HR: 1.70; 95% CI: 1.56–1.82) were associated with an increased risk of HF. The association was also stronger in men than in women. LC, both alcoholic and non-alcoholic, is significantly associated with an increased long-term risk of HF. The association is particularly pronounced in patients with alcoholic cirrhosis and in men. To the best of the authors’ knowledge, this is the first real-world evidence for the positive association between LC and subsequent HF from Europe.

## 1. Introduction

Liver cirrhosis (LC) represents the final stage of chronic liver disease and is a major contributor to global morbidity and mortality. It is responsible for approximately two million deaths annually and accounts for 4% of all global deaths [1]. A nationwide study from Germany revealed a marked increase in the prevalence of both alcoholic and non-alcoholic LC between 2005 and 2018. Additionally, LC was identified as an independent risk factor for in-hospital mortality, increasing the risk sixfold [2].

Traditionally, LC has been associated with hepatic complications such as hepatic encephalopathy and hepatocellular carcinoma. However, it is increasingly recognized as a systemic condition with significant extrahepatic effects [3]. Emerging evidence highlights a complex interaction between liver dysfunction and cardiac pathology, commonly referred to as cardiohepatic syndrome. Structural and functional cardiac abnormalities have been observed in patients with cirrhosis, even in the absence of overt cardiovascular disease [4,5]. One such cardiac complication is cirrhotic cardiomyopathy, which may lead to heart failure (HF) [6].

Although the pathophysiological mechanisms underlying cirrhotic cardiomyopathy and cirrhosis-associated HF have been well studied, large-scale, population-based research on the long-term incidence of clinically diagnosed HF in cirrhotic patients remains limited. Chuzi et al. have retrospectively investigated the association between end stage liver disease (ESLD) and a subsequent HF among 2502 patients with ESLD and 2502 without ESLD in the US. In the multivariable Cox regression analysis, ESLD was significantly associated with a higher risk of incident HF (HR: 4.67; 95% CI: 2.82–7.75) [7].

Previous studies analysed either the link between other liver diseases and HF or between LC and other heart diseases. The meta-analysis of Li et al. including six cohort studies focused on metabolic dysfunction-associated fatty liver disease (MAFLD) and its association with HF risk [8]. After adjusting for multiple confounding risk factors, MAFLD was significantly associated with an increased risk of HF incidence (Hazard Ratio (HR) = 1.36, 95% Confidence Intervals (CI) = 1.16–1.58). Of six cohort studies included in the meta-analysis, in five studies the association between MAFLD and HF was significant with HR values from 1.17 (1.14–1.20) [9] to 1.75 (1.63–1.88) [10].

The study of Hashemi et al. found that patients with LC had a significantly higher prevalence of atherosclerotic cardiovascular disease (ASCVD) events compared to those without cirrhosis, with a HR of 5.73 (95% CI: 2.74–12.72) after adjusting for traditional risk factors [11].

Despite mounting evidence linking liver disease to cardiovascular complications, the long-term risk of clinically diagnosed HF in patients with LC remains underexplored, especially in real-world settings. Notably, most existing studies are based on hospital cohorts or focus on surrogate cardiac markers rather than clinical HF diagnoses. Furthermore, there is a paucity of data from European populations, and no study has systematically compared the differential impact of alcoholic versus non-alcoholic LC on HF risk. Therefore, the present study aimed to evaluate the 10-year incidence of HF in patients with LC, using a large, representative primary care database in Germany. By analyzing subgroups by cirrhosis type, age, and sex, we sought to provide novel insights into high-risk populations and inform future screening and management strategies.

## 2. Methods

### 2.1. Database

This retrospective cohort study utilized data from the Disease Analyzer database (IQVIA), which includes anonymized drug prescriptions, diagnoses, and basic medical and demographic information directly extracted from the computer systems of general practitioners and specialists in Germany [12]. The database comprises information from approximately 3000 office-based physicians and has been shown to be representative of both general and specialized practices across the country [12]. It has been previously used in studies investigating LC [13] and HF [14,15].

### 2.2. Study Population

**Figure 1:**
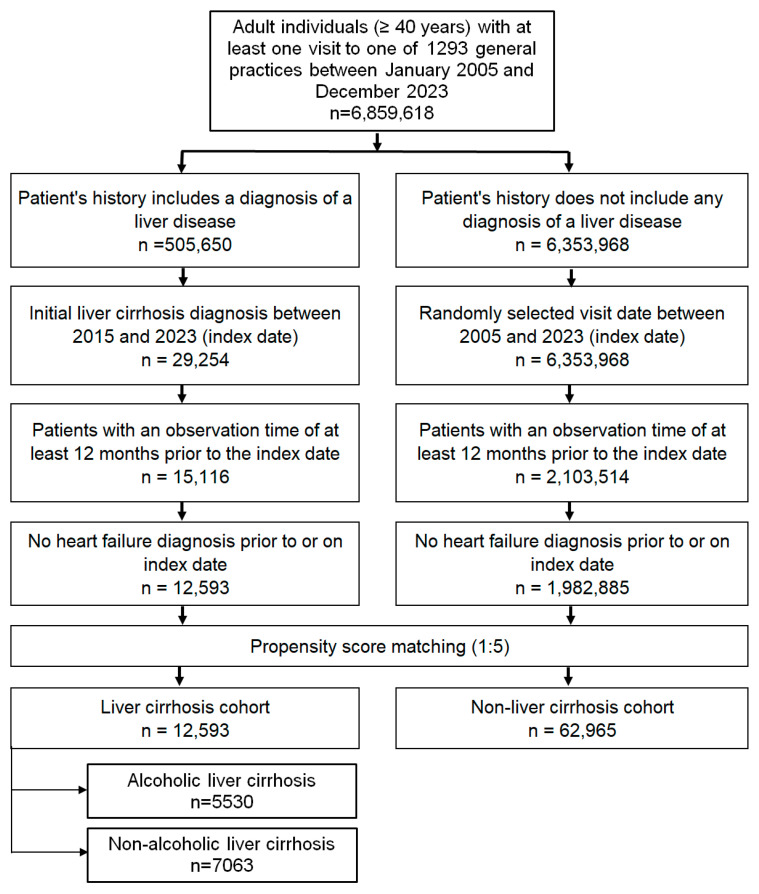
Selection of study patients.

The study population included adults aged ≥ 40 years with a first-time diagnosis of LC (ICD-10 codes: K70.3, K74.3, K74.4, K74.5, K74.6), recorded in 1293 general practices in Germany between January 2005 and December 2023 (index date; see Figure 1). As the database ended in December 2024 and to ensure a minimum of 12 months of follow-up, we defined the index date up to the end of 2023 for patients whose index date occurred that year.

To ensure reliable assessment of comorbidities, only patients with at least 12 months of observation prior to the index date were included. Individuals with a prior diagnosis of HF (ICD-10: I1506–I169, G450) at or before the index date were excluded.

A comparison cohort without liver diseases (ICD-10: K70–K77) was matched to the cirrhosis group using 5:1 nearest-neighbor propensity score matching based on age, sex, index year, average annual consultation frequency, and comorbidities documented within 12 months prior to or on the index date, including obesity (E66), diabetes mellitus (E10–E14), hypertension (I10), dyslipidemia (E78), ischemic heart diseases (I20–I25), and chronic obstructive pulmonary disease (COPD) (ICD-10: J44). For this non-liver disease group, the index date was defined as a randomly selected visit during the study period. Covariate balance between cohorts was assessed using standardized mean differences (SMD), with a value < 0.1 indicating adequate balance.

### 2.3. Study Outcomes

The outcome was the first diagnosis of HF (HF) within 10 years following the index date, analysed in relation to LC status. Alcoholic and non-alcoholic LC were assessed separately. Alcoholic LC was defined as ICD-10: K70.3 or K74.3–K74.6 in combination with a documented history of alcohol dependency (ICD-10: F10). Non-alcoholic LC was defined as K74.3–K74.6 without any recorded diagnosis of alcohol dependency.

### 2.4. Statistical Analyses

The 10-year cumulative incidence of heart failure (HF) was assessed using Kaplan–Meier survival curves, with differences between groups evaluated using the log-rank test. To evaluate the association between LC and incident HF, multivariable Cox proportional hazards models were used. Patients with LC were matched to controls without liver disease using 5:1 nearest-neighbor propensity score matching without replacement and with a caliper of 0.1 standard deviations of the logit of the propensity score. The propensity score was estimated using logistic regression and included the following baseline covariates documented within 12 months prior to or on the index date: age, sex, index year, average annual consultation frequency, obesity (ICD-10: E66), diabetes mellitus (E10–E14), hypertension (I10), dyslipidemia (E78), ischemic heart disease (I20–I25), and chronic obstructive pulmonary disease (COPD; J44). Matching was conducted separately for patients with alcoholic LC and non-alcoholic LC. In the non-LC cohort, the index date was assigned as a randomly selected visit during the study period; the randomization process was stratified to align with the distribution of index years in the LC group to minimize temporal bias. The Cox models were adjusted for medications prescribed within 12 months prior to or on the index date, including diuretics, beta-blockers, calcium-channel blockers, angiotensin-converting enzyme (ACE) inhibitors, angiotensin II receptor blockers (ARBs), lipid-lowering drugs, and sodium-glucose co-transporter 2 (SGLT2) inhibitors. Subgroup analyses were conducted stratified by sex and age categories (<60, 60–69, and ≥70 years). To account for multiple comparisons (six Cox models across different subgroups), we applied a conservative Bonferroni-adjusted threshold for statistical significance of *p* < 0.01. All analyses were conducted using SAS version 9.4 (SAS Institute, Cary, NC, USA).

### 2.5. Ethical Standards

Since only anonymized date were used which could not be traced back to individual persons, the research protocol did not have to be approved by the local ethics committee, and it was not necessary to obtain informed consent from individual patients to participate in the study. This was confirmed by the local ethics committee of the Christian-Albrechts-University (CAU) of Kiel, Kiel, Germany.

## 3. Results

### 3.1. Baseline Characteristics

The final study cohort included 5530 patients with alcoholic LC and 27,650 matched patients without liver disease, as well as 7063 patients with non-alcoholic LC and 35,315 matched patients without liver disease.

Baseline characteristics are presented in Table 1. The mean age was 60.0 years (SD: 10.5) for patients with alcoholic LC and 65.6 years (SD: 11.4) for those with non-alcoholic LC. Women accounted for 30.6% of the alcoholic LC group and 48.1% of the non-alcoholic group. On average, patients had 10 physician visits per year during follow-up. Due to propensity score matching, baseline characteristics were well balanced between patients with and without LC.

Table 2 shows the medications which were prescribed within 12 months prior to or on index date. Diuretics and betablockers were prescribed more frequently in LC patients since other medications were more often prescribed in patients without LC.

### 3.2. Cumulative Incidence of HF

**Figure 2:**
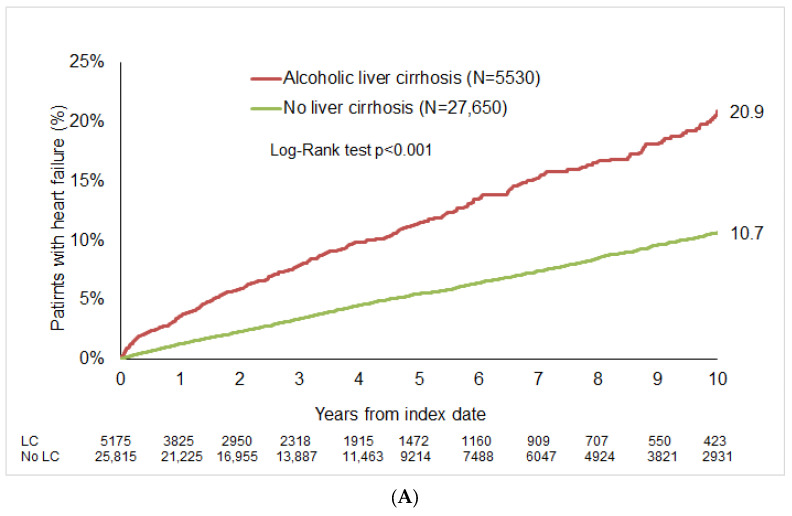

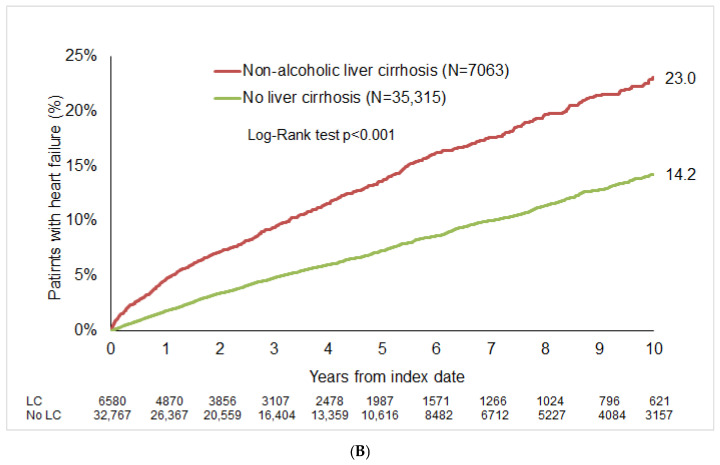
Cumulative incidence of HF in patients with and without LC. (**A**) Alcoholic LC. (**B**) Non-alcoholic liver cirrhosis.

After up to 10 years of follow-up, HF was diagnosed in 20.9% of patients with alcoholic LC compared to 10.3% of the matched cohort (*p*  <  0.001; Figure 2A). In the non-alcoholic LC group, the 10-year cumulative incidence of HF was 23.0%, compared to 14.2% in matched cohort (Figure 2B).

### 3.3. Association Between LC and HF

Cox regression analysis demonstrated a significant association between LC and increased HF risk. Alcoholic LC was associated with an HR of 2.07 (95% CI: 1.85–2.31), and non-alcoholic LC with an HR of 1.70 (95% CI: 1.56–1.82).

Among patients with alcoholic cirrhosis, the association was strongest in those aged < 60 years (HR: 3.54; 95% CI: 2.91–4.29). Men exhibited a higher risk (HR: 2.26; 95% CI: 1.99–2.57) compared to women (HR: 1.63; 95% CI: 1.32–2.03).

Regarding non-alcoholic cirrhosis, the strongest associations were found in patients aged < 60 years (HR: 2.43; 95% CI: 1.97–3.01) and 60–69 years (HR: 2.05; 95% CI: 1.75–2.40), with a lower but still significant association in those ≥70 years (HR: 1.54; 95% CI: 1.37–1.73). The association was also stronger in men (HR: 1.91; 95% CI: 1.71–2.13) than in women (HR: 1.47; 95% CI: 1.29–1.67) (Table 3).

## 4. Discussion

In this large population-based cohort study, we observed that both alcoholic and non-alcoholic LC were significantly associated with an increased risk of subsequent HF over a 10-year follow-up period. This association remained robust across different age groups and was consistently higher in men compared to women. To the best of the authors’ knowledge, this is the first real-world evidence for the positive association between LC and subsequent HF from Europe.

The main result of the present study is the strong association between both alcoholic and non-alcoholic LC and HF which might be explained as following. A substantial proportion of cirrhotic patients experience cardiac dysfunction, including diastolic dysfunction, which is prevalent in 61.9% of cases [16]. This dysfunction can lead to HF, particularly HF with preserved ejection fraction (HFpEF) [17]. Furthermore, LC activates the renin-angiotensin-aldosterone system (RAAS) and sympathetic nervous system, contributing to volume overload and myocardial remodeling—both major risk factors for HF [18]. Moreover, chronic liver disease is associated with ongoing inflammation, which can damage myocardial tissue and impair cardiac function [19].

In the present study, the association between alcoholic LC and HF was slightly stronger than the association between non-alcoholic LC and HF. First, patients with alcoholic cirrhosis are more likely to have heart dysfunction due to direct toxic effects of alcohol on cardiac muscle [20]. Secondly, alcohol contributes to hypertension, arrhythmias, and other cardiovascular problems, which can independently lead to or worsen HF [21,22]. Third, when the heart and liver are both compromised (which often occurs in alcohol-related disease), there is a synergistic worsening of both conditions. Finally, non-alcoholic LC, especially from non-alcoholic steatohepatitis, may be associated with the so called metabolic syndrome (diabetes, obesity, dyslipidemia), which can contribute to diastolic cardiac dysfunction and HFpEF [23,24]. As these disorders are well documented and were included in the matching process for this study, the effect of these disorders when they were available at baseline was eliminated, which could weaken the effect of non-alcoholic LC.

Another important finding of the present work is the stronger association between LC and HF in male than in female patients. On the one side, this gender difference may stem from baseline risk differences. Men generally have higher baseline cardiovascular risk due to factors like greater visceral fat accumulation, higher prevalence of hypertension, and earlier onset of atherosclerosis [25]. Cirrhosis may exacerbate these pre-existing risks more prominently in men. There may also be sex-based differences in diagnosis and treatment patterns, potentially leading to underrecognition or later-stage diagnosis in men, which could worsen outcomes.

Another important finding of this study is the stronger association between LC and HF observed in men compared to women. Several factors may contribute to this difference. From a biological perspective, sex hormones influence cardiovascular physiology and liver disease progression. Estrogen has been shown to exert protective effects on the cardiovascular system by promoting vasodilation, reducing inflammation, and attenuating cardiac remodeling, which may partly explain the lower HF risk observed in women with LC [26,27]. Sociobehavioral factors may also play a role. Men are generally more likely to engage in health-risk behaviors such as heavy alcohol use, smoking, and poor dietary habits—all of which may worsen both hepatic and cardiac function [28]. Together, these biological and behavioral factors likely contribute to the observed sex-related disparities in HF risk among cirrhotic patients.

In the present study, we observed that the association between LC and HF was less strong in patients aged 70+ years. First, this may be a type of selection bias. Many patients with severe cirrhosis and/or alcohol-related disease do not survive into old age. Therefore, people with cirrhosis who live past 70 may represent a healthier or less severely affected subset, making the observed HF risk appear lower [29,30].

Moreover, in older adults, other causes of mortality (e.g., cancer, infections, other organ failure) compete with HF as outcomes. Even if LC still contributes to cardiovascular stress, the overall contribution to HF appears to be diluted because these patients may die from other causes before developing overt HF [31].

Finally, the etiology of cirrhosis differs by age, which might affect the associated HF risk. In younger patients, cirrhosis is more likely due to alcohol or viral hepatitis, both of which can have direct cardiac effects. In older adults, cirrhosis is more often connected to NASH or autoimmune causes, which are less likely to have direct cardiac toxicity [32,33]. Indeed, 18.9% of patients with alcoholic LC but 38.8% of patients with non-alcoholic LC were aged 70+ in this study.

This study has several important limitations that should be acknowledged. First, due to its observational and retrospective design, causal relationships between liver cirrhosis (LC) and heart failure (HF) cannot be firmly established. Although we used rigorous propensity score matching and adjusted for several known confounders, the possibility of residual confounding remains. Second, misclassification of diagnoses is possible since the study relied on ICD-10 coding in routine primary care documentation. While previous validations of the Disease Analyzer database support its reliability, we cannot exclude errors in coding or underreporting—particularly in the case of alcohol use disorders. Some patients with alcoholic cirrhosis may have been misclassified as having non-alcoholic cirrhosis due to stigma-related underreporting or lack of documentation. Third, several clinically relevant variables were not available in the dataset. These include echocardiographic parameters, HF subtype or severity (e.g., NYHA class), cardiac biomarkers, and details on liver disease stage (e.g., MELD or Child-Pugh scores). The absence of such information limits our ability to explore the pathophysiological link between LC and HF more precisely or to adjust for disease severity. Fourth, key lifestyle and behavioral factors such as smoking status, alcohol consumption patterns, physical activity, diet, and socioeconomic status were not recorded. These factors may independently influence both the risk of developing cirrhosis and HF and could therefore act as unmeasured confounders. Their absence may have introduced bias in the estimated associations. Finally, although medication use was adjusted for, we did not have data on treatment adherence, dosages, or longitudinal medication changes. These aspects could also have affected HF risk over time. Taken together, these limitations emphasize the need for future prospective studies with richer clinical and behavioral data to validate and extend our findings.

The findings of this study have important clinical implications. First, clinicians should be aware that both alcoholic and non-alcoholic liver cirrhosis is associated with an increased risk of subsequent heart failure, particularly in male and younger patients. This suggests a need for routine cardiovascular risk assessment and longitudinal cardiac monitoring in patients with cirrhosis, even in the absence of overt cardiac symptoms. Second, early identification of cirrhotic cardiomyopathy or subclinical cardiac dysfunction may enable timely intervention, potentially improving long-term outcomes. Incorporating cardiac evaluations—such as echocardiography and natriuretic peptide measurement—into the standard management of cirrhosis may be warranted, especially in high-risk subgroups. Lastly, these results underscore the importance of interdisciplinary collaboration between hepatologists and cardiologists. Given the systemic nature of liver cirrhosis and its strong cardiohepatic interactions, a more integrated care model may help in optimizing outcomes and reducing morbidity associated with heart failure in this population.

## 5. Conclusions

LC, both alcoholic and non-alcoholic, is significantly associated with an increased long-term risk of HF. The association is particularly pronounced in patients with alcoholic cirrhosis and in men. These findings highlight the need for further studies to explore underlying mechanisms and guide early cardiovascular monitoring in cirrhosis patients.

## Figures and Tables

**Table 1:** Baseline characteristics of the study sample (after 1:5 propensity score matching).

Variable	Proportion Among Patients with Alcoholic LC (N, %) N = 5530	Proportion Among Patients Without LC (N, %) N = 27,650	SMD	Proportion Among Patients with Non-Alcoholic LC (N, %) N = 7063	Proportion Among Patients Without LC (N, %) N = 35,315	SMD
Age (Mean, SD)	60.0 (10.5)	59.9 (10.7)	0.008	65.6 (11.4)	65.8 (11.6)	−0.020
Age < 60	2811 (50.8)	14,177 (51.3)		2211 (31.3)	11,065 (31.3)	
Age 60–69	1691 (30.6)	8238 (29.8)		2150 (30.4)	10,555 (29.9)	
Age 70+	1028 (18.6)	5235 (18.9)		2702 (38.3)	13,695 (38.8)	
Female	1693 (30.6)	8564 (31.0)	−0.004	3400 (48.1)	17,136 (48.5)	−0.004
Male	3837 (69.4)	19,086 (69.0)		3663 (51.9)	18,179 (51.5)	
Number of physician visits per year during the follow-up (Mean, SD)	9.9 (4.2)	10.0 (4.1)	−0.030	9.9 (4.2)	10.0 (4.1)	−0.033
Obesity	649 (11.7)	3053 (11.0)	−0.007	1007 (14.3)	4691 (13.3)	−0.010
Diabetes mellitus	1503 (27.2)	7302 (26.4)	−0.008	2513 (35.6)	12,241 (34.7)	−0.009
Hypertension	3124 (56.5)	15,647 (56.6)	0.001	4130 (58.5)	20,663 (58.5)	0.000
Dyslipidemia	1622 (29.3)	8008 (29.0)	−0.004	2217 (31.4)	11,185 (31.7)	0.003
Ischemic heart diseases	719 (13.0)	3484 (12.6)	−0.004	1230 (17.4)	5819 (16.5)	−0.009
COPD	987 (17.9)	4671 (16.9)	−0.010	1061 (15.0)	5017 (14.2)	−0.008

Proportions of patients in N, % given, unless otherwise indicated. SD: standard deviation. SMD- Standardized mean difference.

**Table 2:** Medications prescribed within 12 months prior to or on the index date.

Variable	Proportion Among Patients with Alcoholic LC (N, %) N = 5530	Proportion Among Patients Without LC (N, %) N = 27,650	*p* Value	Proportion Among Patients with Non-Alcoholic LC (N, %) N = 7063	Proportion Among Patients Without LC (N, %) N = 35,315	*p* Value
Diuretics	2293 (41.5)	41,192 (15.2)	<0.001	2851 (40.4)	7291 (20.7)	<0.001
Betablockers	2199 (39.8)	8163 (29.5)	<0.001	2882 (40.8)	12,170 (34.5)	<0.001
Calcium-channel blockers	764 (13.8)	7873 (17.6)	<0.001	1303 (18.5)	7479 (21.2)	<0.001
ACE inhibitors	1505 (27.2)	8253 (29.9)	<0.001	1922 (27.2)	11,200 (31.7)	<0.001
Angiotensin II receptor blockers	671 (12.1)	5434 (19.7)	<0.001	1371 (19.3)	7762 (22.0)	<0.001
Lipid lowering drugs	902 (16.3)	6369 (23.0)	<0.001	1534 (21.7)	9657 (27.4)	<0.001
SGLT2 inhibitors	86 (1.6)	662 (2.4)	<0.001	244 (3.5)	948 (2.7)	<0.001

Number and proportions of patients in N, %; *p* value was estimated using chi squared test.

**Table 3:** Association between LC and subsequent HF diagnoses in patients followed in general practices in Germany (multivariable Cox regression models).

	Alcoholic LC		Non-Alcoholic LC	
Patient Subgroup	HR (95% CI) *	*p* Value	HR (95% CI) *	*p* Value
Total	2.07 (1.85–2.31)	<0.001	1.70 (1.56–1.82)	<0.001
Age < 60	3.54 (2.91–4.29)	<0.001	2.43 (1.97–3.01)	<0.001
Age 60–69	2.13 (1.75–2.58)	<0.001	2.05 (1.75–2.40)	<0.001
Age 70+	1.43 (1.18–1.72)	<0.001	1.54 (1.37–1.73)	<0.001
Female	1.63 (1.32–2.03)	<0.001	1.47 (1.29–1.67)	<0.001
Male	2.26 (1.99–2.57)	<0.001	1.91 (1.71–2.13)	<0.001

* adjusted for medications prescribed within 12 months prior to or index date including diuretics, betablockers, calcium-channel blockers, angiotensin-converting enzyme (ACE) inhibitors, angiotensin II receptor blockers, lipid lowering drugs, and sodium-glucose transport protein 2 (SGLT2) inhibitors.

## Data Availability

Data were obtained from IQVIA and are available upon reasonable request with the permission of IQVIA. Restrictions apply due to data protection requirements.
